# A mixed-methods model for healthcare system responsiveness to public health: insights from Iranian experts

**DOI:** 10.1186/s12961-025-01295-y

**Published:** 2025-02-21

**Authors:** Hooman Khanpoor, Ahad Alizadeh, Omid Khosravizadeh, Mohammad Amerzadeh, Sima Rafiei

**Affiliations:** 1 Student Research Committee, School of Health, Qazvin University of Medical Sciences, Qazvin, Iran; 2 Social Determinants of Health Research Center, Research Institute for Prevention of Non-Communicable Diseases, Qazvin University of Medical Sciences, Qazvin, Iran; 3 Non-Communicable Diseases Research Center, Research Institute for Prevention of Non-Communicable Diseases, Qazvin University of Medical Sciences, Qazvin, Iran; 4 Health Services Management Department, School of Public Health, Qazvin University of Medical Sciences, Shahid Bahonar Blv, 3419759811 Qazvin, Iran

**Keywords:** Health systems, Public health, Health services accessibility, Healthcare quality, Access, Evaluation

## Abstract

**Background:**

Responsiveness is a critical dimension of public health, focussing on how health systems address the needs, preferences and expectations of the population. It plays a central role in improving and maintaining the population’s health by ensuring timely, equitable and patient-centred healthcare services.

**Objective:**

In this study, we developed a mixed-methods model to assess healthcare system responsiveness from a public health perspective, integrating the insights of Iranian experts. The model identifies key factors such as intersectoral collaboration, health equity and community partnerships, which are essential for enhancing system responsiveness and ultimately improving health outcomes.

**Method:**

In this study, conducted in 2024, we developed a mixed-methods model for assessing healthcare system responsiveness from a public health perspective, integrating the insights of Iranian experts. R software version 3.2.4 and the lavaan package were used for statistical analysis, considering the significance level of 0.05.

**Results:**

On the basis of the literature review, the main components of health systems’ responsiveness in the public health domain were extracted. The qualitative content analysis induced three different themes that affect health, which included payment mechanism (two subcategories of budget and incentive system), social determinants of health (three subcategories of intersectoral collaboration, community partnership and equity in health) and quality (three subcategories of timely provision of healthcare services, need-based service delivery and continuity of care). Finally, using structural equation modelling (SEM), a system of variables with causal relationships was developed. We found a statistically significant direct effect on intersectoral collaboration, health equity, payment mechanisms, timely delivery of services and need-based service provision. The strongest association was found for the payment system (*β* = 1.023, *P* ≤ 0.05). Model fit indices showed adequate fit.

**Conclusions:**

Our developed model underscores the need for a comprehensive approach to healthcare system responsiveness, particularly focussing on public health services as foundational strategies for achieving universal health coverage. The results of our study revealed that a well-structured payment system and incentive mechanisms are critical for motivating healthcare professionals to deliver high-quality, timely and need-based services, ensuring sustainability in care provision. Beyond financial incentives, our model highlights the importance of health equity, intersectoral collaboration and community partnerships, which were identified as key drivers of responsiveness in healthcare systems. The findings indicate that fostering these elements not only strengthens healthcare delivery, but also supports the adaptation of services to meet diverse population needs effectively. In addition, the study emphasizes the innovative role of intersectoral collaboration in enhancing primary healthcare, which requires commitment across healthcare and non-health sectors. Our model introduces the concept of integrating community participation and resource allocation strategies into the healthcare system, thereby enhancing responsiveness. These strategies are anticipated to improve key health outcomes, such as life expectancy and maternal and child health indicators, by establishing robust healthcare networks that are more attuned to the health needs of the population.

## Background

Health systems play a crucial role in improving and maintaining individuals’ health across their lifespan. According to the WHO, health systems should aim to achieve three fundamental goals: health promotion within communities, financial protection and responsiveness and people-centred care [1, 2].

Responsiveness contributes to a health system’s capacity to meet the authentic needs and expectations of a population and the way they are treated in the face of the health system [3]. One of the essential aspects of responsiveness relates to effective relationships between various actors within the health system and people’s involvement in setting priorities for their health [4–7]. The health system’s responsiveness includes a more inclusive concept within which everyone has a fair opportunity to achieve optimal health regardless of gender, race, ethnicity, religion, disability, identity or socioeconomic status [8]. Health system responsiveness, as defined by the WHO in the 2000 World Health Report, refers to “the health system’s ability to meet the legitimate expectations of the population regarding non-health aspects of their interactions with the system” [1]. These non-health expectations include factors such as dignity, autonomy, confidentiality, prompt attention, social support, basic amenities and the choice of healthcare provider. Unlike health outcomes, which are directly related to the quality and accessibility of care, responsiveness addresses the way individuals are treated within the healthcare system, emphasizing the importance of respect and consideration in healthcare interactions. This concept highlights the crucial role of healthcare providers not only in delivering medical care, but also in ensuring that patients feel valued and respected throughout their healthcare experience [1]. However, public health responsiveness extends beyond patient–provider interactions to encompass the health system’s capacity to proactively address population-level health needs, ensure equitable access to preventive services and adapt to the emerging public [9].

The responsiveness of health systems varies significantly across countries, with notable differences in how well health systems meet the expectations of their populations. According to the WHO’s assessment, countries such as the United States, Switzerland, Luxembourg, Denmark, Germany, Japan, Canada, Norway, the Netherlands and Sweden were recognized as having some of the most responsive health systems [1]. These countries tend to have well-established health systems with high levels of resource availability, strong infrastructure and a cultural emphasis on patient-centred care. In contrast, lower-income or underdeveloped nations often struggle with resource constraints, which can hinder their ability to meet population expectations for responsiveness. Factors such as economic conditions, healthcare infrastructure and cultural norms play a significant role in shaping the responsiveness of health systems. Understanding these variations is essential for developing context-specific models that can enhance the responsiveness of health systems worldwide, including those in low-resource settings [9, 10].

Despite several studies investigating the determinants of health system responsiveness across countries, there has been little study on the factors of responsiveness in the public health domain [1, 9–15]. On the basis of existing literature, several theoretical frameworks were proposed to situate the healthcare system responsiveness within a broader structure [15, 16]. Most of them are built on interaction between the patients and the health system while utilizing health services [16]. These frameworks cover different aspects of user satisfaction with medical aspects of healthcare [9, 16].

Several other theories focus on a specific aspect of responsiveness, such as patient communication skills, timely and relevant information or provider accountability [17, 18]. However, an inclusive framework demands the consideration of public interest towards the responsiveness of a healthcare system, not just patient interests that access available services. The latter perspective might exclude certain population groups and disregard their viewpoints concerning the critical determinants of responsiveness [19, 20]. Likewise, some nonmedical factors entitled “social determinants of health” significantly affect a population’s health outcomes. These factors are constituted by numerous direct and indirect relationships between interrelated variables such as lifestyle factors and socioeconomic, cultural and environmental conditions that lead to several health problems [21, 22].

The use of participatory methods in determining the contributing factors to the responsiveness of healthcare systems can reveal interrelated variables to be used in formulating a comprehensive model [23]. Thus, to cover the research gap and develop an inclusive model using different stakeholders’ points of view, we conducted a mixed-method research study to develop a conceptual framework for responsiveness from the public health perspective, building upon existing theoretical frameworks.

## Theoretical foundation of the conceptual model

The development of our public health responsiveness model builds upon existing frameworks of health system responsiveness, particularly the WHO’s 2000 Health System Performance Framework, which identifies responsiveness as a key function of health systems along with improving health outcomes and financial protection [1]. However, while the WHO’s model primarily focusses on individual-level experiences within healthcare services, our study expands this perspective by integrating population-level determinants of responsiveness within public health.

In addition, our model aligns with social determinants of health (SDH) frameworks, which emphasize the role of socioeconomic, political and environmental factors in shaping health outcomes [24]. Unlike traditional responsiveness models that primarily assess patient-provider interactions, our conceptual model incorporates equity, intersectoral collaboration and community participation as essential dimensions that drive responsiveness in the public health domain.

Furthermore, our model is grounded in systems thinking, recognizing the interconnected nature of health determinants and the need for multisectoral strategies [25]. The inclusion of incentive mechanisms, funding allocation and governance structures ensures a holistic understanding of how responsiveness can be institutionalized within public health policies rather than remaining a passive service attribute.

By bridging these theoretical perspectives, our model introduces an integrated and action-oriented approach that extends beyond individual healthcare interactions to emphasize preventive care, equity-driven service provision and collaborative governance, making it uniquely positioned to address the challenges of public health responsiveness in diverse healthcare settings.

Our model introduces a novel, inclusive framework for assessing healthcare system responsiveness, addressing gaps in existing theories that often focus narrowly on specific aspects such as patient–provider communication, timely information or provider accountability [17, 18]. While these approaches are valuable, they tend to emphasise individual patient interests and may exclude the perspectives of broader population groups, particularly those whose needs are not adequately captured by conventional healthcare models. By incorporating the public interest and acknowledging the viewpoints of diverse stakeholders, our model overcomes these limitations. This approach ensures that critical determinants of responsiveness, such as social determinants of health, are integrated into the framework. Social determinants, which include lifestyle, socioeconomic, cultural and environmental factors, significantly impact population health outcomes [21, 22]. However, these factors are often overlooked in traditional models that focus primarily on healthcare delivery rather than the broader context of health disparities. Through a mixed-methods approach, our study integrates both qualitative and quantitative data to develop a conceptual framework that reflects the diverse needs and experiences of the population. This inclusive, stakeholder-driven model is positioned to offer a more comprehensive and adaptable solution for enhancing healthcare system responsiveness, particularly in the context of public health, where a holistic understanding of health determinants is essential.

## Materials and methods

In conducting this mixed-methods study, we followed the guidelines outlined in the Guidance for Reporting Mixed-Methods Studies [26], which provides a comprehensive framework for combining qualitative and quantitative research approaches in health systems research. This guideline recommends clear articulation of the research questions, the integration of both data types and a transparent explanation of the data collection and analysis process to ensure rigour and reproducibility. By adhering to these established guidelines, we aimed to ensure the rigour, validity and reproducibility of our study’s findings, while maintaining transparency throughout the research process.

### Study design and participants

This study utilized a mixed-method design combining both quantitative and qualitative approaches to develop a responsive model in public health. The research was conducted in three distinct phases: a literature review, qualitative expert panel consultations and quantitative data collection using confirmatory factor analysis (CFA). Utilising a mixed-methods approach facilitated the integration of qualitative data from expert panels and quantitative data through CFA to develop a comprehensive model for healthcare system responsiveness.

In the quantitative phase, a sample size of 208 participants was determined on the basis of a recommended ratio of 15–20 participants per model variable, considering the eight key components in the structural equation model (SEM). Data were collected through a survey assessing the impact of various health system components on responsiveness. Confirmatory factor analysis (CFA) was employed to examine the relationships between the variables and validate the model. The SEM process involved five stages: (1) formulation of the initial conceptual model, (2) data collection, (3) construction of variable matrices, (4) estimation and evaluation of model fit using goodness-of-fit indices and (5) presentation of a final validated responsiveness model.

### Study phases

#### Literature review (phase 1)

A systematic literature review was conducted to identify key factors influencing health system responsiveness. The search strategy included databases such as Web of Science, Scopus, ScienceDirect, OVID, CINHAL, EBSCO, Google Scholar, Iranmedex, SID and Irandoc.

*Search strategy:* the following keywords and Boolean operators were used: “health system” AND “responsiveness” AND “public health” AND “modeling”. Filters were applied to select articles published between 2000 and 2022 in English and Persian.

Inclusion criteria:

- Empirical studies, reviews and theoretical frameworks related to public health responsiveness
- Studies focusing on health policy, service quality and equity
- Publications providing quantitative or qualitative insights into public health modelling

Exclusion criteria:

- Studies focussed solely on patient satisfaction without a broader system perspective
- Clinical trials unrelated to public health service delivery
- Articles without full-text access or insufficient methodological details

A total of 17 articles met the inclusion criteria and were reviewed in-depth. Extracted variables were compiled into a structured table for expert evaluation.

#### Qualitative phase (phase 2)

A Delphi method was employed to refine the extracted factors through expert consensus. Experts were selected on the basis of their expertise in healthcare management, public health policy-making, health promotion and preventive care systems.

*Sampling method:* A purposive sampling technique was used to recruit 12 experts from universities, public health departments and healthcare policy organizations. Inclusion criteria included: (1) a minimum of 10 years of experience in healthcare policy, (2) prior research or policy-making contributions in public health and (3) willingness to participate in multiple rounds of discussion.

The Delphi process included two rounds:

*Round 1:* experts assessed the relevance, transparency and applicability of the identified variables.

*Round 2:* a revised version was sent back for reassessment, ensuring consensus was reached before finalizing the conceptual model.

#### Quantitative phase (phase 3)

In this phase, confirmatory factor analysis (CFA) was used to examine the relationships between the identified variables and construct the final responsiveness model. The CFA was conducted using R software (version 3.2.4), and the sample size was determined on the basis of recommendations for SEM.

### Expert panel characteristics

The expert panel consisted of 12 participants, whose demographics are presented in Table 1. The panel members were professionals with varying expertise, including health management, policy-making and health promotion. Their experience was vital for ensuring that the conceptual model was both comprehensive and relevant to the local context.

**Table 1:** Characteristics of expert panel participants

Characteristics		*N*	*N*%
Gender	Male	4	33.4
	Female	8	66.6
Age	30–40	3	33.4
	40–50	5	41.6
	≥ 50	4	8.4
Job title	Network management and health promotion	2	16.6
	Disease prevention and control management	1	8.3
	Environmental and occupational health management	2	16.6
	Family and school health management	3	25.3
	Mental and social health management	2	16.6
	Oral and dental health management	1	8.3
	Nutritional health management	1	8.3
Length of service, years	< 10	2	16.6
	10–20	7	58.3
	≥ 20	3	25.1

#### Hypotheses

On the basis of the literature review and expert panel input, the following hypotheses were tested in the quantitative phase:

- H1: Intersectoral collaboration significantly impacts the health system’s responsiveness in public health.
- H2: Community partnership significantly impacts the health system’s responsiveness in public health.
- H3: Equity in health significantly impacts the health system’s responsiveness in public health.
- H4: Allocated budget to the health sector significantly impacts the health system’s responsiveness in public health.
- H5: Incentive mechanisms and types of payment to healthcare professionals significantly impact the health system’s responsiveness in public health.
- H6: Provision of need-based services significantly impacts the health system’s responsiveness in public health.
- H7: Provision of timely services significantly impacts the health system’s responsiveness in public health.
- H8: Continuation of care significantly impacts the health system’s responsiveness in public health.

### Data collection

In the qualitative phase, a questionnaire was designed on the basis of insights from the literature review and expert panel feedback. The questionnaire included two sections: demographic information and a series of questions designed to assess the factors influencing the responsiveness of the public health system. A five-point Likert scale (1 = very low, 5 = very high) was used to measure the perceived importance of each factor. The final tool was reviewed for content validity by the expert panel before data collection began.

### Data integration

Integration occurred at two levels: during the data collection phase, in which expert input informed the development of the survey, and during analysis, in which qualitative findings were used to refine the CFA model.

### Quality and rigour

To ensure validity, the content validity of the survey was assessed through expert feedback, while CFA assessed the construct validity of the relationships between variables.

### Sample source and data analysis

The sample for the quantitative phase consisted of employees working in public health departments and healthcare centres affiliated with the university. Inclusion criteria required participants to have relevant knowledge and experience in health policy-making, primary care services or public health service delivery. A total of 208 participants were included in the study, ensuring an adequate sample size for the CFA analysis. Descriptive statistics were used to analyse demographic data and the responses to the questionnaire.

Given that the objective of this study was to test specific hypotheses regarding the relationships between key determinants of health system responsiveness rather than to explore an unknown factor structure, CFA was chosen over exploratory factor analysis (EFA). The constructs and variables were initially derived from a literature review and further refined through expert panel evaluations to ensure their conceptual validity. Therefore, CFA was utilized to validate the predefined structure of the model and assess the relationships between the identified factors, in alignment with the study’s theoretical framework.

CFA was employed to examine the relationships between the identified variables, and model fit was assessed using goodness-of-fit indices.

In the data analysis phase, descriptive and inferential statistics were used to evaluate the data. Descriptive analysis provided insights into the sample’s characteristics, while confirmatory factor analysis was conducted to validate the proposed model and assess the strength and direction of the relationships between the variables. All analyses were performed using R software (version 3.2.4), with a significance level of 0.05.

## Results

### Components of health system responsiveness in public health

A review of 17 articles published between 2000 and 2022 identified 19 components influencing health system responsiveness in the public health domain, as presented in Table 2. Amongst the components, factors such as quality of healthcare services, community partnership and health equity emerged as the most frequently discussed in the literature.

**Table 2:** The components of the responsiveness model from the public health domain

Components
Quality in the provision of healthcare services
Health equity and considering the health needs of different population groups
Public participation in the provision of public health services
Allocated budget for the health sector
Payment system and incentive mechanisms for healthcare professionals to address health issues
Intersectoral collaboration to ensure a comprehensive health package for community members
Provision of healthcare services based on the population’s health needs
Timely provision of services
Continuity of care
Socioeconomic status of the population
Deprivation
Demographic characteristics such as age, disability, pregnancy, childbirth, race, religion and gender
Establishing communication
Autonomy
Access to healthcare services
Efficiency
Managing cultural change
Knowledge and experience of service providers
Patient-centred and interpersonal care

### Conceptualization of components and related dimensions

**Fig. 1:**
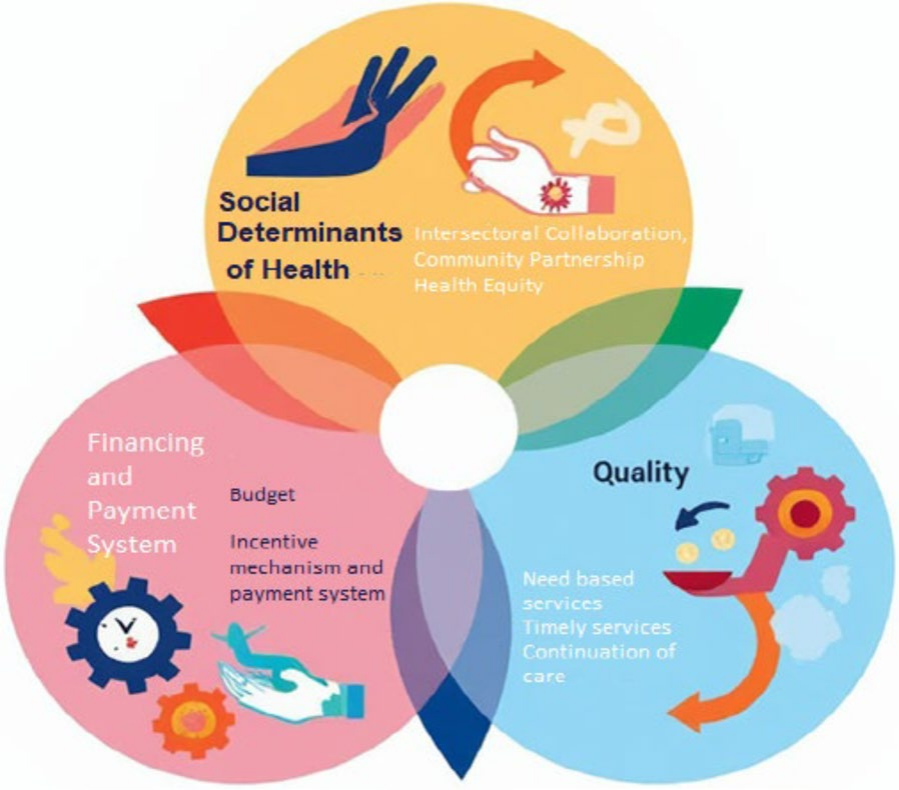
Conceptual model of responsiveness in the public health

During the expert panel session, the identified components were further refined and redefined for clarity and applicability in the public health context. These components were organized into broader dimensions, which were subsequently conceptualized within the proposed model as illustrated in Fig. 1.

### The effects of different components on the responsiveness of the public health model

**Fig. 2:**
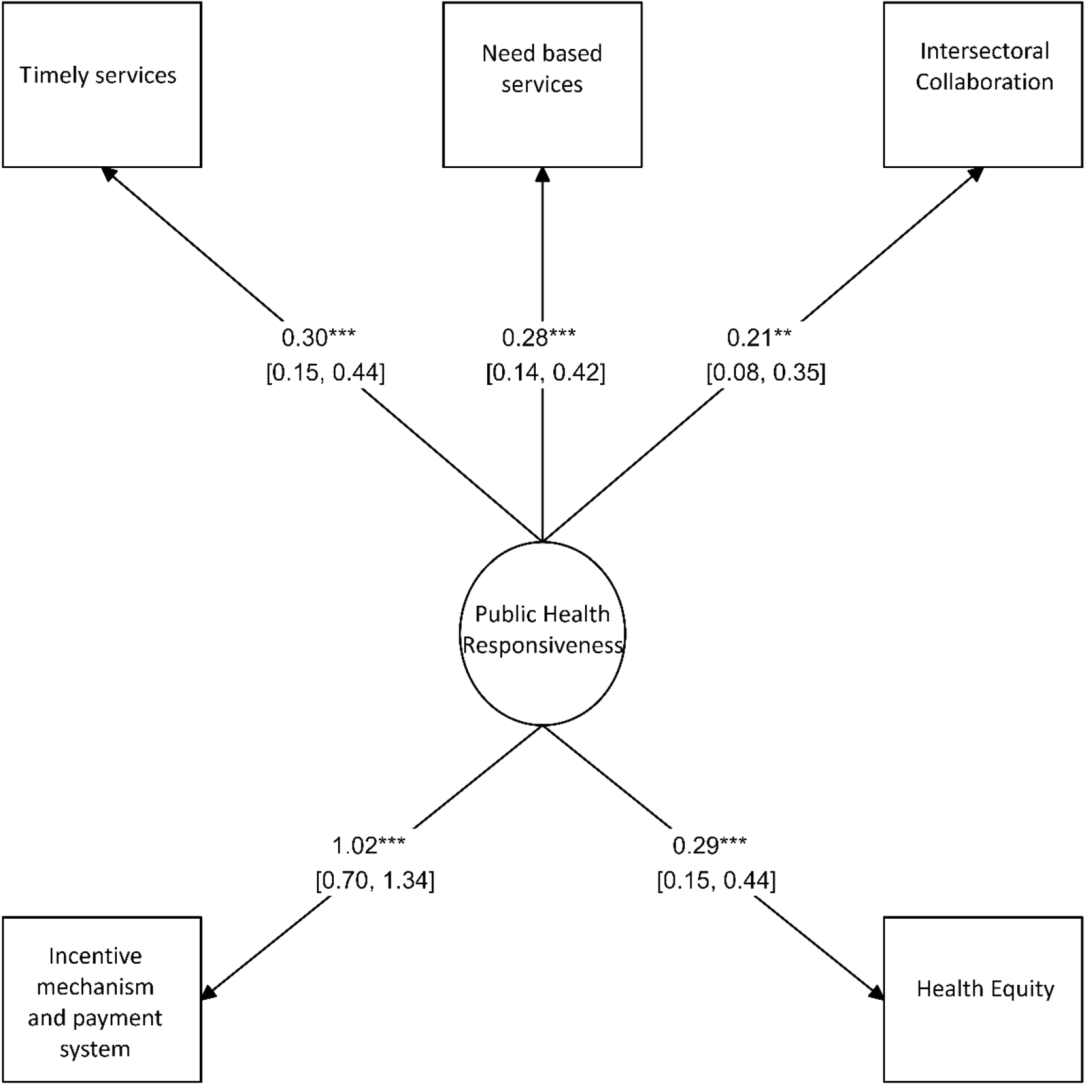
The impact of different components on the responsiveness of the healthcare system

On the basis of the standardized coefficients shown in Fig. 2 and presented in Table 3, several components demonstrated statistically significant positive effects on the responsiveness of the public health system. The components of payment and incentive systems, timely provision of health services, health equity, need-based service provision and intersectoral collaboration exhibited strong and significant contributions to improving responsiveness.

**Table 3:** The impact of health components on the responsiveness of the health system in the public health domain

Component	Standard coefficient	Non-standardized coefficient	95% confidence intervals	*P*-value
Timely provision of healthcare needs	0.299	1	–	–
Need-based services	0.278	1.592	0.687–2.497	0.001
Incentive mechanism and payment system	1.023	6.11	1.602–10.617	0.008
Health equity	0.295	1.661	0.747–2.575	0.001
Intersectoral cooperation	0.212	1.596	0.516–2.677	0.004

An increase of one standard deviation in each of these components resulted in a significant increase in health system responsiveness in the public health domain.

The structural model exhibited acceptable fit, as evidenced by a goodness-of-fit index (GFI) of 0.973, an adjusted goodness-of-fit index (AGFI) of 0.918 and a lower bound of the root mean square error of approximation (RMSEA) of 0.058.

## Discussion

This study aimed to identify the dimensions that significantly impact the healthcare system’s responsiveness, focussing on the public health domain.

### Model innovation and differences from existing models

One of the key innovative aspects of this study lies in the development of a comprehensive, multidimensional responsiveness model for public health systems that integrates both the social determinants of health and health system functionality. The integration of intersectoral collaboration and need-based service provision within the same model represents a novel contribution to the field. While many existing models focus on individual factors such as access to healthcare services or health equity, our model uniquely considers the interplay between these diverse factors, emphasizing how they collectively enhance responsiveness.

Moreover, our model incorporates an adaptive approach, where components can be refined and adjusted through expert panel feedback, ensuring its applicability to local contexts and evolving health challenges. This adaptive feature allows for greater flexibility and relevance in applying the model across different countries or health systems with varied priorities.

Another key difference lies in the inclusivity of stakeholder perspectives. By consulting a broad range of experts across various public health and policy sectors, our model offers a more holistic view compared with existing models, which often limit their focus to healthcare providers or policy-makers alone.

With this approach, critical factors of responsiveness in the healthcare system include reorganizing primary care to meet community needs, improving the quality of services in various centres, transforming education in different areas of primary healthcare, motivating healthcare professionals at various levels and addressing their needs, such as payment systems and incentives for healthcare providers to address health-related issues and promote community health. Amongst the most critical areas for organizing a responsive healthcare system are ensuring access to appropriate appointments with physicians, the attitudes and behaviours of healthcare workers, system alignment with patient needs, coordination and support for continuous and ongoing care and attention to intersectoral coordination with all sectors of the community to achieve comprehensive population health. According to the study findings, the responsiveness components of the healthcare sector are categorized into three main dimensions: attention to social determinants of health, financing and payment mechanisms and quality of provided services. Our proposed framework also emphasized the importance of promoting justice in healthcare and meeting the needs of different social groups, as some groups may require additional support. Therefore, the discussion of health system responsiveness in healthcare is crucial for policy-making in the health sector to improve health status and reduce inequalities, especially about the role of social determinants. Amongst these components, the quality of healthcare services based on the population’s needs, public participation in the provision of services and equity in health played a significant role in the responsiveness of the healthcare system. One of the critical components of social determinants of health is intersectoral collaboration. Multisectoral collaboration can potentially drive transformative change for health and sustainable development by encouraging novel collaboration and knowledge exchange forms. It can also create opportunities for innovative approaches and learning, leading to positive outcomes for health and the long-term sustainability of services [27, 28]. Therefore, it is suggested that future trends in public administration should shift towards fostering more collaboration and partnerships among governance agencies, citizens and various stakeholders instead of solely prioritizing responsiveness [29].

The second component of social determinants of health is a community partnership. Effective partnership of the community in the provision of health services can enhance the health and well-being of the population through addressing existing challenges due to the governance and management, facilitating the achievement of goals and objectives and tackling social determinants of health and improving them to control the diseases effectively [30]. In line with these findings, a study conducted by Mirzoev and Kane found that the responsiveness of health systems is affected by the dynamics and interactions between the individuals as service users and the health system, which can potentially provide valuable insights for assessing the current situation and implementing appropriate interventions to enhance health outcomes [31].

The last component in the social determinants of health is health equity. In agreement with our findings, a study by Marmot revealed that achieving health equity necessitates addressing the underlying causes of diseases by evaluating the circumstances in which individuals grow, live, work and age. Targeting these factors can improve health outcomes for all community members [24]. Health equity refers to the absence of systematic disparities in the health condition of a society with varying levels of social advantage or disadvantages. It also emphasizes fairness and equality in health outcomes, regardless of an individual’s social circumstances [24].

Another key responsiveness component in our study is the payment system and incentive mechanisms, which include two subcomponents. In a study by Robone et al., increasing healthcare expenditures per capita and expanding the provision of services through effective collaboration between the nongovernmental sector and private providers can significantly enhance the responsiveness of a health system [32]. Another study found a significant relationship between higher patient-perceived responsiveness in primary care systems and increased health expenditure manifesting in physician’s remuneration through the capitation system [33]. In addition, allocating more financial resources to primary healthcare and public health services can increase the accountability of a health system and improve transparency of governmental providers in costing their budget. Studies have confirmed that by incorporating financial measurements into the evaluation mechanisms of healthcare systems, it becomes easier to track and evaluate the allocation of resources, assess the efficiency and effectiveness of public expenditures and ensure the transparency and accountability of financial transactions [34]. Such a system would ultimately lead to improved governance, better decision-making and increased public trust in the management of public funds. Similar studies also revealed a positive correlation between corporate responsiveness and improved financial performance of a healthcare system [32–34].

Another component of responsiveness in our study is quality, which encompasses need-based services, timely services and continuation of care. Compassionate objectives create a pattern of responsiveness in the relationships between providers and service recipients. Appropriate communication will be fostered through developing empathy and mutual understanding, resulting in reciprocal interactions between different healthcare system partners [35]. A study by Valentine et al. revealed that the most critical factors of nonclinical quality of care are effective communication, immediate attention to people’s needs and treating them with respect and dignity [27]. As defined by the WHO, the concept of responsiveness can be applied to emphasize the importance of considering people’s needs and expectations while offering them healthcare services, preserving their dignity and promoting their autonomy in seeking desirable treatment approaches. By gauging service quality from the users’ perspectives, this approach enables a comprehensive assessment of healthcare delivery [1].

Similarly, another study highlighted continuity of care for terminally ill patients, which entails addressing their physical, emotional, socioenvironmental and spiritual challenges. This comprehensive approach is grounded in both scientific competence and compassionate attitudes, ensuring that appropriate care is provided in line with the population’s diverse needs. Continuation of care in inpatient settings has shown significant advantages for patients, particularly in reducing the frequency and duration of hospitalizations for individuals with a history of frequent readmissions [36].

## Study strengths and limitations

One of the significant strengths of this study is the broad applicability of the developed responsiveness model, particularly in varying geographical and resource contexts, including underdeveloped and developing countries. The multidimensional nature of the model, which incorporates factors such as health equity, timely service provision, community participation and intersectoral collaboration, ensures that it can be adapted to diverse healthcare systems, regardless of their stage of development.

In underdeveloped countries, where healthcare systems may face significant resource constraints, our model remains highly relevant. By prioritizing the need-based provision of services, the model emphasizes the importance of targeting healthcare delivery to the most vulnerable populations. This approach is particularly critical in resource-limited settings, where focussing on the most urgent health needs – such as maternal and child health, infectious diseases and primary care services – can significantly improve overall health system responsiveness.

In addition, the intersectoral collaboration component of the model aligns well with the needs of underdeveloped countries. In these settings, health systems often suffer from fragmented services and a lack of coordination between sectors such as education, agriculture, water sanitation and healthcare. Our model encourages cross-sector partnerships, which can enhance service delivery, optimize resources and improve health outcomes in ways that are both cost-effective and sustainable.

The community participation aspect of the model is another crucial feature for underdeveloped countries, where public health systems can often be disconnected from the communities they serve. By involving community members in health decision-making and service provision, the model fosters greater engagement, trust and accountability, all of which are vital in low-resource settings. This participatory approach can help ensure that services are culturally relevant and tailored to the unique needs of different population groups.

Moreover, the model’s emphasis on equity in health ensures that even underdeveloped countries, with their diverse populations, can address disparities in health access and outcomes. By focusing on equitable distribution of resources and services, the model promotes fairness, which is essential for countries where marginalized or underserved groups often face barriers to accessing care.

In developing countries, which are often in the process of strengthening their health systems, the responsiveness model can act as a tool for guiding policy development and optimizing health service delivery. For example, the model can help identify gaps in healthcare services and propose targeted interventions to enhance access and quality.

However, this study has some limitations that should be acknowledged. First, the sample size was relatively small, which might have limited the generalizability of our findings to a larger population. Although the sample size was small, we addressed this limitation by ensuring the participants were selected through a rigorous random sampling process. We also conducted statistical tests to assess the significance of our findings and reported effect sizes to provide a sense of the practical importance of the results. Another limitation is the potential for response bias, as participants might have provided socially desirable responses or might not have felt comfortable sharing their authentic experiences or opinions. We employed various measures during data collection to address the potential for response bias.

Finally, the study was conducted over a relatively short time frame owing to resource constraints, which might have limited our ability to capture long-term effects or changes over time. Recognizing these limitations is essential for interpreting and generalizing the findings of our study. We focused on the immediate impacts and changes observed during the study period to mitigate this limitation. We suggested that future research with extended time frames could provide a more comprehensive understanding of the phenomenon under investigation.

Another limitation is that formal content validity assessment using the content validity index (CVI) and content validity ratio (CVR) was not conducted. Instead, content validity was ensured through expert panel evaluations and iterative refinements before applying CFA. While this approach provided a systematic review of the questionnaire items, future studies should incorporate quantitative measures such as CVI and CVR to further strengthen the validity of the instrument. Despite this limitation, CFA provided strong statistical evidence for the construct validity of the model, supporting its applicability in assessing health system responsiveness.

## Implications for further studies

Incorporating artificial intelligence (AI) into the responsiveness model could facilitate automated decision-making on the basis of ongoing health system performance data, improving efficiency and enhancing responsiveness. For example, AI could be used to identify areas where service provision is lagging behind, such as delays in health service delivery, and recommend corrective actions on the basis of historical data patterns. Moreover, the model could support predictive analytics, allowing health systems to forecast future health challenges and proactively adjust resources and policies to address emerging needs. By integrating these modern technological solutions, the model would not only provide a comprehensive understanding of health system responsiveness, but also offer practical tools for enhancing it in a rapidly changing healthcare landscape.

## Conclusions

The comprehensive and adaptable nature of this model makes it suitable for diverse health system contexts, whether in underdeveloped, developing or more developed countries. By considering different dimensions of public health responsiveness, the model can be fine-tuned to fit local priorities, healthcare challenges and resource availability.

In wealthier countries, where healthcare infrastructure is often more robust, the model’s focus on quality of care, efficiency and patient-centred services would provide valuable insights for optimizing existing systems. In contrast, for underdeveloped countries, the model’s emphasis on equitable access to care, intersectoral collaboration and community participation can help address fundamental challenges related to infrastructure, access and health equity.

Ultimately, the model’s design ensures that it can be applied across a spectrum of health system settings, demonstrating its global relevance and potential impact. Its flexibility and adaptability make it a powerful tool for policy-makers, health system planners and public health experts worldwide, regardless of their country’s developmental status.

## Data Availability

The datasets used and/or analysed during the current study are available from the corresponding author on reasonable request. The entire dataset is in the Farsi language. The data can be available in English for the readers and made available from the corresponding author on reasonable request.‎
